# Resveratrol Induces Oxidative Stress and Downregulates GPX4 and xCT to Activate the Ferroptosis Pathway for Anti-Bladder Cancer Organoids

**DOI:** 10.7150/jca.109350

**Published:** 2025-06-09

**Authors:** Xiang Chen, Jun-Lin Lu, Hong Li, Guang-Yao Liu, Ting-Ting Li, Kang-Hua Xiao, Hai-Shan Ye, Sheng Li, Xu Chen, Jia Liu

**Affiliations:** 1 School of Medicine, South China University of Technology, Guangzhou 510006, China.; 2 Department of Urology, Sun Yat-sen Memorial Hospital, Sun Yat-sen University, Guangzhou, Guangdong 510120, China.; 3 Guangdong Provincial Key Laboratory of Malignant Tumor Epigenetics and Gene Regulation, Sun Yat-Sen Memorial Hospital, Sun Yat-Sen University, Guangzhou, Guangdong 510120, China.; 4 BioMed Laboratory, Guangzhou Jingke Biotech Group, Guangzhou 510005, China.; 5 Liaoning Laboratory of Cancer Genetics and Epigenetics, College of Basic Medical Sciences, Dalian Medical University, Dalian 116044, China.

**Keywords:** resveratrol, bladder cancer, organoid, oxidative stress, ferroptosis

## Abstract

**Purpose:** Resveratrol (RES) exhibits promising anti-bladder cancer (BC) effects. It causes cell death of BC cell lines and xenografts, but comprehensive analyses are required concerning oxidative status in RES-treated BC cells and its relevance with cell death patterns, and especially the efficacy of BC individuals.

**Methods:** Two human BC cell lines (T24 and UM-UC-3) were cultured under 2D and 3D conditions to determine RES IC_50_ values and 100 μM RES was thus used as a working concentration to treat 18 cases of BC-derived organoids (BCDOs). To observe the experimental results, this study will assess the oxidative stress status of bladder cancer cells, the corresponding potential cell death patterns, and the extent of inhibition by death inhibitors.

**Results:** It revealed growth suppression of 10 RES-treated BCDOs (55.56%), accompanied by reduced mitochondrial potential and increased Reactive Oxygen Species (ROS) levels. Lipid peroxidation and downregulation of GPX4 and xCT, key antioxidant molecules of ferroptosis, were also found in RES-sensitive BC cells, and the inhibition rate of ferroptosis inhibitors increased to 22.57% of BCDOs. In addition, the supplementary experiment also indicated that it may be related to apoptosis and autophagy pathways. The above cellular and molecular alterations were not distinct in RES-insensitive BCDOs.

**Conclusion:** RES possesses promising inhibitory effects on either BC cell lines or a large part (10/18) of BCDOs via enhancing oxidative stress and triggering the ferroptosis pathway or more. These findings underscore anti-BC properties of RES and its role as a potential personalized treatment option.

## Introduction

Bladder cancer (BC) is one of the most common cancers worldwide and its incidence rate in males is approximately four times higher than that in females 1. Non-muscle-invasive bladder cancer (NMIBC) accounts for 75% of BCs, which are usually treated by transurethral resection supplemented by postoperative bladder instillation chemotherapy 2. Five-year recurrence-free survival (RFS) rates of NMIBC is over 30%, while 21% of patients advance to muscle-invasive bladder cancer (MIBC) due to drug resistance and recurrence 3. The standard treatment for MIBC is cisplatin-based systemic neoadjuvant chemotherapy followed by radical cystectomy 4. However, the outcome of the current chemotherapy strategy is not optimistic due to high toxicity and drug resistance 5. It would be worthwhile to explore novel anti-BC agent(s) with low toxicity and better efficacy.

Natural bioactive products including resveratrol (RES) have become a cutting-edge research trend because of their anti-tumor effects and lesser toxicity 6. A body of *in vitro* evidence reveals that RES exerts inhibitory effects on bladder cancers 7, 8. Even though, RES has not yet been used in anticancer therapy, because of its low bioavailability when administered systemically 9. Therefore, organ-targeted RES delivery may overcome this therapeutic dilemma and the bladder is a suitable organ for intravesical drug instillation. Our previous study proved that this RES administration approach was effective to suppress mouse orthotopic bladder cancers without causing local and systemic toxic effect on the treatment mice 10, which might be due to the efficient metabolic machinery for RES was operated in normal cells rather than the malignant ones 11, 12. Consequently, RES possesses a biphasic dose-dependence effect: a cytoprotector as an antioxidant at a low concentration, and a pro-oxidant at high concentration 13-15. We have substantiated this assertion in several experimental cancer systems 16, 17. It has been increasingly recognized that targeting-induced generation of reactive oxygen species (ROS) can be utilized in cancer management because this approach can activate the cell death pathways including apoptosis, autophagy, necrosis, and/or ferroptosis 18. Ferroptosis is a novel form of programmed cell death primarily induced by the accumulation of iron-dependent ROS and excessive lipid peroxidation 19. However, a comprehensive investigation into the death mechanism induced by RES-mediated oxidative stress in BC cells has not yet been available. An address of this issue would provide further experimental evidence for the application of RES in BC management, especially in intravesical instillation administration.

The data concerning the inhibitory effects of RES on BCs were largely obtained from BC lines 20, 21 and/ or xenograft models formed by BC lines or tumor tissues 10. Both experimental systems are prone to cellular and genetic variations after multiple passages and undergo significant disparities with the actual situation of cancer patients 22, 23. To achieve a closer approximation of the clinical efficacy of experimental findings, it is imperative to validate the reliability of the *in vitro* results using organoids derived from BC patients. To reach that goal, our current study analyzes the efficacy of RES to 3D-cultured BC cell lines and a cohort of BC-derived organoids (BCDOs) and its relevance with oxidative stress-caused cell death.

## Material and Methods

### Cell lines and cell culture

Human bladder transitional cell carcinoma cell lines (T24 and UM-UC-3) were obtained from American Type Culture Collection (Manassas, VA, United States) 24, 25. T24 cells were cultured in RPMI1640 medium (Gibco, Thermo Fisher Scientific, Suzhou, China) and UM-UC-3 cells were cultured in DMEM (Gibco, Thermo Fisher Scientific, Suzhou, China) supplemented with 10% fetal bovine serum (Gibco Life Science, Grand Island, NY, USA) and 1% penicillin-streptomycin (Gibco Invitrogen Corp., Grand Island, NY, USA) under standard condition at 37 ºC in a humidified atmosphere of 5% CO_2_ and 95% air.

### Cell spheroids models

T24 and UM-UC-3 cell lines were cultured in growth medium until reaching 80% confluency, digested with Trypsin-EDTA (Gibco, Thermo Fisher Scientific, Suzhou, China), resuspended in growth medium, and counted with a hemocytometer. Spheroids models were generated from the two cell lines by embedding cell suspension with a 1:2 volume Matrigel matrix (Corning, NY, USA) and at a density of 400~600 cells/10 μL. The mixtures were seeded into 48 well plates or 300~400 cells/6 μL into 96 well plates and incubated for one week at 37 ºC in a humidified atmosphere of 5% CO_2_ and 95% air.

### BC organoid modeling

BC organoids (BCDOs) were cultured from surgical samples exclusively obtained from the operating room at Sun Yat-sen Memorial Hospital, Sun Yat-sen University. The research protocol was subject to a thorough review and granted approval by the hospital's esteemed medical ethics committee (Ethical code number: SYSKY-2023-396-01). The steps of BC tissue isolation, digestion, filtration, and organoid culture can be performed as described in the previous articles 25, 26. Fresh BC tissues were washed 6-8 times in PBS (Gibco, NY, USA) containing 100U/ml penicillin (Gibco, NY, USA) and 100 μg/ml streptomycin (Gibco, NY, USA). Subsequently, the tissues were cut into small fragments (about < 0.5mm), and incubated with 1 mL TrypLE (Gibco, NY, USA) at 37 ºC for 30 min. After digestion, the isolated cells were filtered through a 70 μm cell filter, and centrifuged at 1000 rpm for 5 minutes following separation. After centrifugation, the cells were mixed with BCDO culture medium and mixed with a ratio of 1:2 volume in Matrigel matrix (Corning, NY, USA). About 15 μL drops were embedded in the preheated 48-well plate. After the drops were solidified, 250 μL of BCDO culture medium was added to each well, followed by cultivation in an incubator at 37 ºC, 5% CO_2_, and 95% air humidity condition. The culture medium was replaced every 2 days. The BCDO culture medium consisted of DMEM/F12 (Gibco, Thermo Fisher Scientific, Suzhou, China), 10% fetal bovine serum (Gibco Life Science, Grand Island, NY, USA), 100 μg/mL Noggin (MedChemExpress, NJ, USA), 10 μM/L ROCK inhibitor Y-27632 (MedChemExpress, NJ, USA), 100U/ml penicillin (Gibco, NY, USA) and 100μg/ml streptomycin (Gibco, NY, USA).

### RES treatment

RES (Sigma-Aldrich, St. Louis, MO, USA) was dissolved in DMSO (Sigma-Aldrich, St. Louis, MO, USA) to prepare stock solutions at concentrations of 200 mM/L and 100 mM/L. T24 and UM-UC-3 were cultured under 2D and 3D conditions and then treated with various concentrations (25 μM, 50 μM, 75 μM, 100 μM, 150 μM, 200 μM) of RES (2D for 48h; 3D for 48 and 96h) for determining RES IC_50_ via CCK-8 (for 2D), CCK-3D (for 3D) viability and EdU proliferation assays as described below. The cell lines and BCDOs were then treated with RES according to the results of IC50 determination.

### Inhibitor treatments

Ferrostain-1 (MedChemExpress, NJ, USA), a potent and selective inhibitor of ferroptosis, its storage concentration (dissolved in DMSO) is 10 mM/L and the concentration used is 0.5 μM. Z-VAD-FMK (MedChemExpress, NJ, USA), a pan caspase inhibitor, was dissolved in DMSO to prepare stock solutions at concentrations of 10 mM/L. After testing for toxicity and in combination with RES, 0.5 μM was used as the final concentration. Chloroquine (MedChemExpress, NJ, USA), an autophagy inhibitor, was also dissolved in DMSO to prepare stock solutions at concentrations of 25 mM/L, After the toxicity test, the concentration used is 0.5 μM. The inhibition of cell death was observed using these death inhibitors in combination with 100 μM RES. CCK8 was used to detect the activity of T24 cells in each group, and Calcein/PI count was used to evaluate BCDOs groups.

### Calcein AM/PI cell viability assay

Detection of cell morphology and viability was performed using the Calcein AM/PI cell viability assay kit (Beyotime, Shanghai, China), referred to the previous article. The viable cells are specifically labeled with Calcein AM, resulting in a green fluorescent signal, while nonviable or dead cells are selectively labeled with PI, generating a red fluorescence. Briefly, the cells, spheroids, and organoids samples were incubated with both Calcein AM, PI, and Hoechst 33342 (Beyotime, Shanghai, China) solution for 30 minutes. Afterward, the images of the cells' viability were captured using a fluorescent microscope (Nikon, ECLIPSE NI-U), and red/green cell counts were used to quantify the degree of cytotoxicity exerted by RES on cells and organoids.

### CCK-8 viability assay

The cell viability of T24 and UM-UC-3 under 2D cultured conditions was assessed using the CCK-8 (Meilunbio, Dalian, China). Approximately a density of 4 ×10^3^ cells/ well was added into a 96-well plate. The different concentrations of RES were treated for 48h. Afterward, CCK-8 solution 10 μL/100 μL was added into a well and incubated for 1h at 37 ℃ before testing the absorbance at the wavelength of 450 nm.

### CCK-3D viability assay

Cell viability in T24 and UM-UC-3 formed spheroids was detected by the CCK-3D (Beyotime, Shanghai, China). As described above, the cell suspension with Matrigel matrix (Corning, NY, USA) mixture was aliquoted into the 96-well plate at 300~400 cells/6 μL per well for a week. The spheroids were incubated with the medium containing different concentrations of RES for 48h and 96h. 10 μL/100 μL of CCK-3D liquid was then added to each of the wells for 1h at 37 ºC. The results were collected by a microplate reader (TECAN, infinite F50) by reading the absorbance at the wavelength of 450 nm.

### IC_50_ evaluation

The absorbance values detected through CCK-8 (Meilunbio, Dalian, China) or CCK-3D (Beyotime, Shanghai, China) were subtracted by the blank control values and normalized. The normalized values were obtained through this analysis. Nonlinear regression analysis was conducted using GraphPad Prism 9.0 software, employing the “log(inhibitor) vs normalized response— Variable slope” models. The IC_50_ values and graphical representations of the results were obtained through this analysis.

### EdU proliferation detection

For 5-ethynyl-2'-deoxyuridine (EdU), a thymidine analog that incorporates into DNA during replication and is widely used to detect proliferating cells, the EdU-594 Kit (Beyotime, Shanghai, China) was employed to evaluate the proliferation status of the cells 27. The cells and spheroids were treated with different concentrations of RES for 48h and the BCDOs were treated with 100 μM RES for 96h. Before the last time point, all the groups were labeled with 5-ethenyl-2'-deoxyuridine (EdU) for 8h and then were incubated with Click Addictive solution in the dark for 30 minutes at 25 ºC. Then Hoechst 33342 (Beyotime, Shanghai, China) was used to stain the cell nucleus. Afterward, the images of EdU and nucleus staining were captured by microscope (Nikon, ECLIPSE NI-U).

### Mitochondria staining and fluorescence analysis

Mito-Tracker Red CMXRos (Beyotime, Shanghai, China) is a cell-permeant fluorescent dye that accumulates in mitochondria based on their membrane potential and is widely used for mitochondrial labeling 28-30. T24 and UM-UC-3 cells were treated with 100 μM RES for 48h and the BCDOs for 96h. Then all the samples were incubated with the Mito-Tracker Red CMXRos and Hoechst 33342 (Beyotime, Shanghai, China) solution for 30 minutes in the dark at indoor temperature. The acquisition of images was carried out using a fluorescence microscope (Nikon, ECLIPSE NI-U), followed by the semi-quantitative analysis of the fluorescence intensity using Image J software (NIH, USA).

### ROS level detection

The ROS level was assessed using a reactive oxygen species assay kit (Beyotime, Shanghai, China). DCFH-DA lacks fluorescence itself and can penetrate cell membranes, hydrolyzed by intracellular esterases, and then oxidized by intracellular ROS to generate fluorescent DCF, allowing for the measurement of ROS levels. The cells and BCDOs were treated with 100 μM RES for 0h, 6h, 12h, 24h, and 48h, while the positive control group was treated with 50 μg/mL rosup for 30 minutes. All the samples were stained with 10 μM DCFH-DA in serum-free DMEM (Gibco, Thermo Fisher Scientific, Suzhou, China). The samples stained *in situ* with DCFH-DA and Hoechst 33342 (Beyotime, Shanghai, China), and collected at each of the time points were observed and photographed using a fluorescence microscope (Nikon, ECLIPSE NI-U). The fluorescent images were semi-quantitatively analyzed using Image J software (NIH, USA).

### TUNEL apoptosis assay

Detection of cellar apoptosis was performed using a TUNLE assay kit (Beyotime, Shanghai, China). The 3'-OH exposed during the DNA breakage can be labeled with green fluorescence probe fluorescein-conjugated dUTP catalyzed by terminal deoxynucleotidyl transference (TdT). To assess apoptosis, the cells, and BCDOs were washed twice with PBS, fixed in absolute ethanol, and permeabilized with 0.3% Triton X-100. Then the prepared TUNEL solution was added and incubated in the dark for 1 hour. Lastly, the cells and BCDOs were stained Hoechst 33342 (Beyotime, Shanghai, China) for 30 minutes, and images of TUNEL and Hoechst staining were captured using a fluorescence microscope (Nikon, ECLIPSE NI-U) to observe the cell apoptosis.

### Measurement of lipid peroxidation levels

Measurement of lipid peroxidation was performed using the C11 BODYPI 581/591 lipid peroxidation probe (Maokang Biotechnology, Shanghai, China). The C11 BODYPI 581/591 lipid peroxidation probe possesses lipophilic characteristics and is not spontaneously displaced from the lipid bilayer. Upon lipid peroxidation, a transition from a reduction (red fluorescence) to an oxidation (green fluorescence) is observed. The cells and BCDOs were washed twice with PBS and incubated with C11 BODYPI 581/591 (5 μM) and Hoechst 33342 (Beyotime, Shanghai, China) for 1 hour in the dark at 25 ºC. The images of lipid peroxidation were observed using a fluorescence microscope (Nikon, ECLIPSE NI-U). Fluorescent intensity was measured by ImageJ software (NIH, USA), and the results were indicated as the degree of lipid peroxidation by the ratio values of oxidation/reduction (green/red).

### Immunohistochemical staining

For BCDOs, they were fixed using a 4% paraformaldehyde solution, followed by dehydrating, embedding, sectioning, dewaxing, rehydrating, and retrieving antigens treatments. The samples were treated with 0.3% Triton-100 (Sigma-Aldrich, St. Louis, MO, USA), followed by two washes with PBS and 3% H_2_O_2_, blocked with BSA at 37 ºC, and incubated with the appropriately diluted primary antibody overnight at 4 ºC. The colorimetric staining was performed using DAB (BOSTER, Main St, Fullerton, USA). Rabbit anti-P63 (Abcam, Cambridge, MA, USA), Mouse anti-CK7 (Abcam, Cambridge, MA, USA), Rabbit anti-Ki67 (Abcam, Cambridge, MA, USA), Rabbit anti-GATA-3 (Abcam, Cambridge, MA, USA). Nuclei were counterstained with hematoxylin. Staining results were evaluated based on the intensity of staining (weak, moderate, or strong) and the distribution of positive cells. At least three random fields per sample were assessed.

### Immunofluorescence staining

The cells and BCDOs for immunofluorescence staining were washed three times with PBS, permeabilized using 0.3% Triton X-100, and then incubated overnight at 4 ºC with the diluted primary antibody, followed by 488-conjugated goat IgG (Proteintech, Chicago, USA) or 594-conjugated Mouse IgG (Proteintech, Chicago, USA). Afterwards, Hoechst 33342 (Beyotime, Shanghai, China) was used for nuclear staining. The images of immunofluorescence staining and nucleus staining were captured by microscope (Nikon, ECLIPSE NI-U). Mouse anti-GPX4 (proteintech, Wuhan, China), rabbit xCT/SLC7A11 (proteintech, Wuhan, China), rabbit anti-LC3α/β (Wanleibio, Shenyang, China) and rabbit anti-Beclin1 (Wanleibio, Shenyang, China).

### Fluorescence image quantification

Images were acquired using a Nikon EClIPSE NI-U microscope under standardized conditions. Fluorescence intensity (Mean/IntDen) was quantified using ImageJ (NIH, USA): entire images for cell monolayers (background-substracted) and manually delineated ROIs for organoids. Data were normalized to image area (cell monolayers) or cluster count (organoids) and analyzed using specific test (GraphPad Prism v9.0; p < 0.05).

### Statistical analyses

The analyses of the results were performed using one-way ANOVA and t-test in GraphPad Prism 9.0 (San Diego, USA). The experimental data were expressed as mean ± standard deviation. All the experiments were repeated at least three times.

## Result

### Establishment of BC experimental models

**Figure 1:**
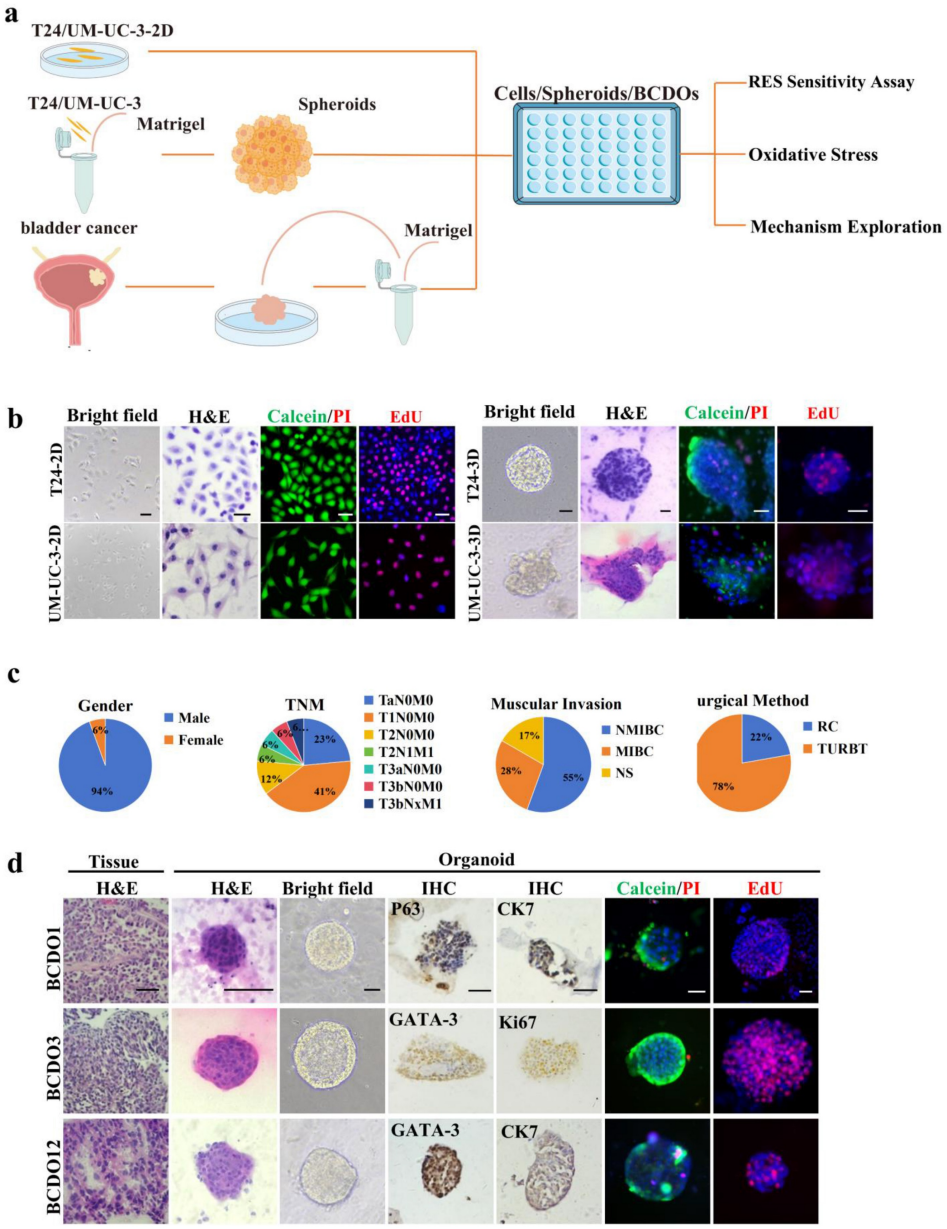
** Experimental procedure and establishment of drug sensitivity systems.** (a) The overview of the experimental process of sensitivity detection of RES for the three models and mechanism exploration on BC cells. (b) 2D and 3D-cultured T24 and UM-UC-3. (c) Basic clinical information of BC patients. NMIBC: Non-muscle-invasive bladder cancer; MIBC: Muscle-invasive bladder cancer; NS: not seen muscular layer; RC: Radical cystectomy; TURBT: Transurethral resection of Bladder Tumor. (d) HE of primary BCDOs and their parental tissues; bright-field images and BC biomarker oriented immunohistochemical staining (P63, CK7, GATA-3, KI67) of BCDOs. Scale bar: 50 μm.

The experimental procedure is depicted in Figure 1a. T24 and UM-UC-3 cells formed spheroids with diameters ranging from150-200 μm after one week of 3D culture (Figure. 1b). BCDO models were established using the surgical samples of 18 high-grade urothelial carcinomas, and the basic clinical information of patients is presented in Figure 1c and Table S1. Pathological similarities (morphology, nuclear-to-cytoplasmic ratio dysregulation, atypia) between BCDOs and their parental tumors, and the molecular characteristics of BC cells observed in BCDOs through immunohistochemical staining for BC-associated biomarkers (GATA-3, P63, KI67, and CK7) 31 were shown in Figure 1d.

### Time-related IC_50_ of RES to 2D- and 3D-cultured BC cells

**Figure 2:**
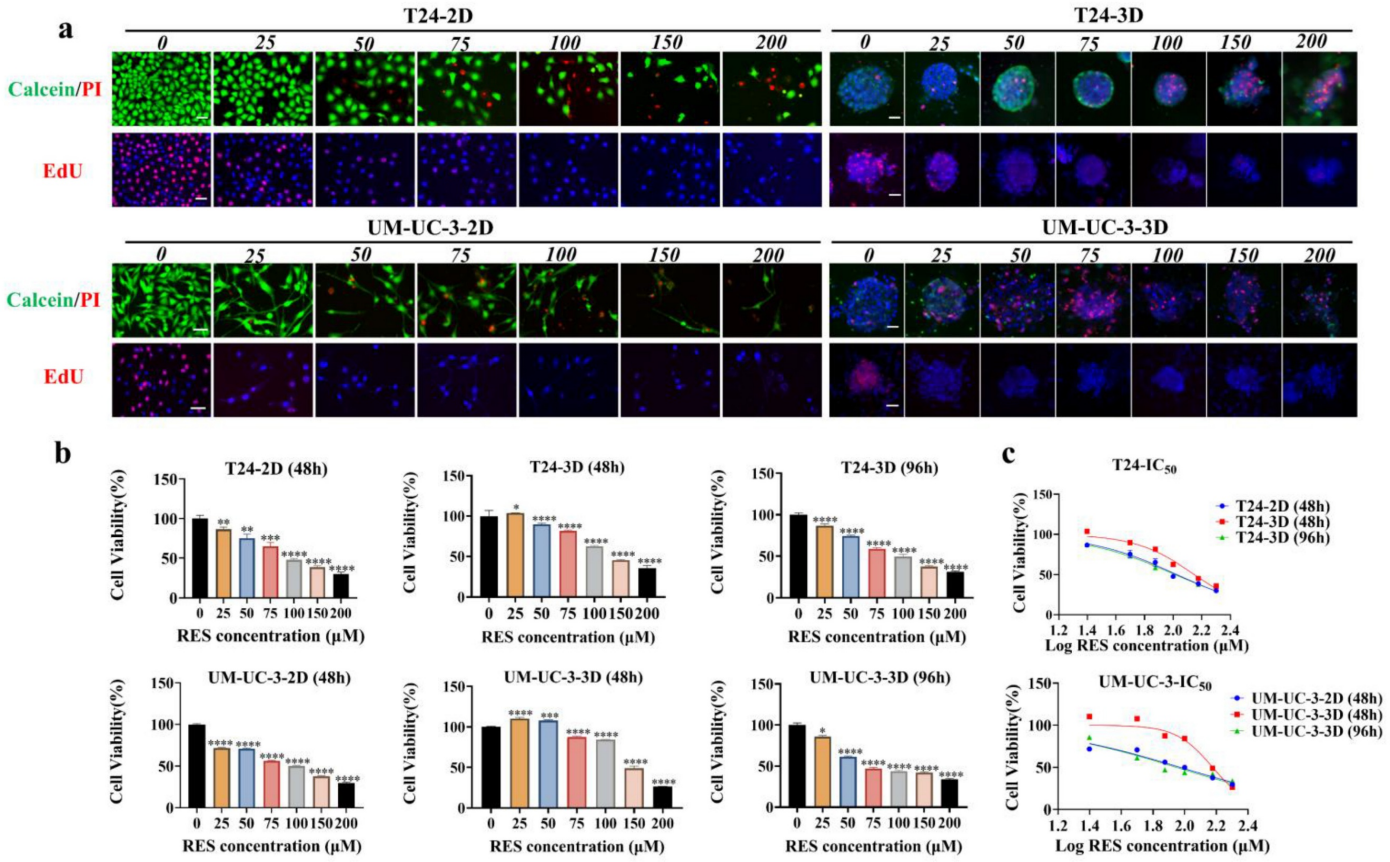
** Inhibitory effects and IC_50_ values of RES on BC cells.** (a) The images of Calcein/PI and EdU staining of the T24 and UM-UC-3 cells and spheroids with different concentrations (0, 25, 50, 100, 150, and 200 μM) RES treatment for 48h or 96h. The scale bar: 50 μm. (b) The results of cell viability of both cell lines and spheroids were measured by CCK-8 assay. (c) The curve graphically represents the IC_50_ values.

The viability and proliferation experiments (Figure 2a and Figure S1) showed that, as concentration of RES increased, T24 and UM-UC-3 cells in 2D culture underwent growth suppression and structural alteration in dose- and time-related fashions. Interestingly, a biphasic effect of RES was observed at 48 hours, where 25 μM RES stimulated cell viability in the 3D cultured spheroids, while higher concentrations led to a reduction in cell viability. This phenomenon is consistent with previous studies that have reported similar biphasic responses to RES in various cancer cell models, as mentioned in the introduction. Similar drug response patterns were also observed in 3D cultured spheroids (Figure 2a and Figure S1). The CCK-8 assay demonstrated that the proliferative activity of BC cells decreased gradually with an increase in RES concentration (Figure 2b). The IC_50_ values and visual images for cell lines and spheroids are presented in Table S2 and Figure 2c. These results suggest that the effective working concentration for RES treatment in the 3D model was set at 100 μM with a treatment time of 96h.

### Different response of BCDOs to RES

**Figure 3:**
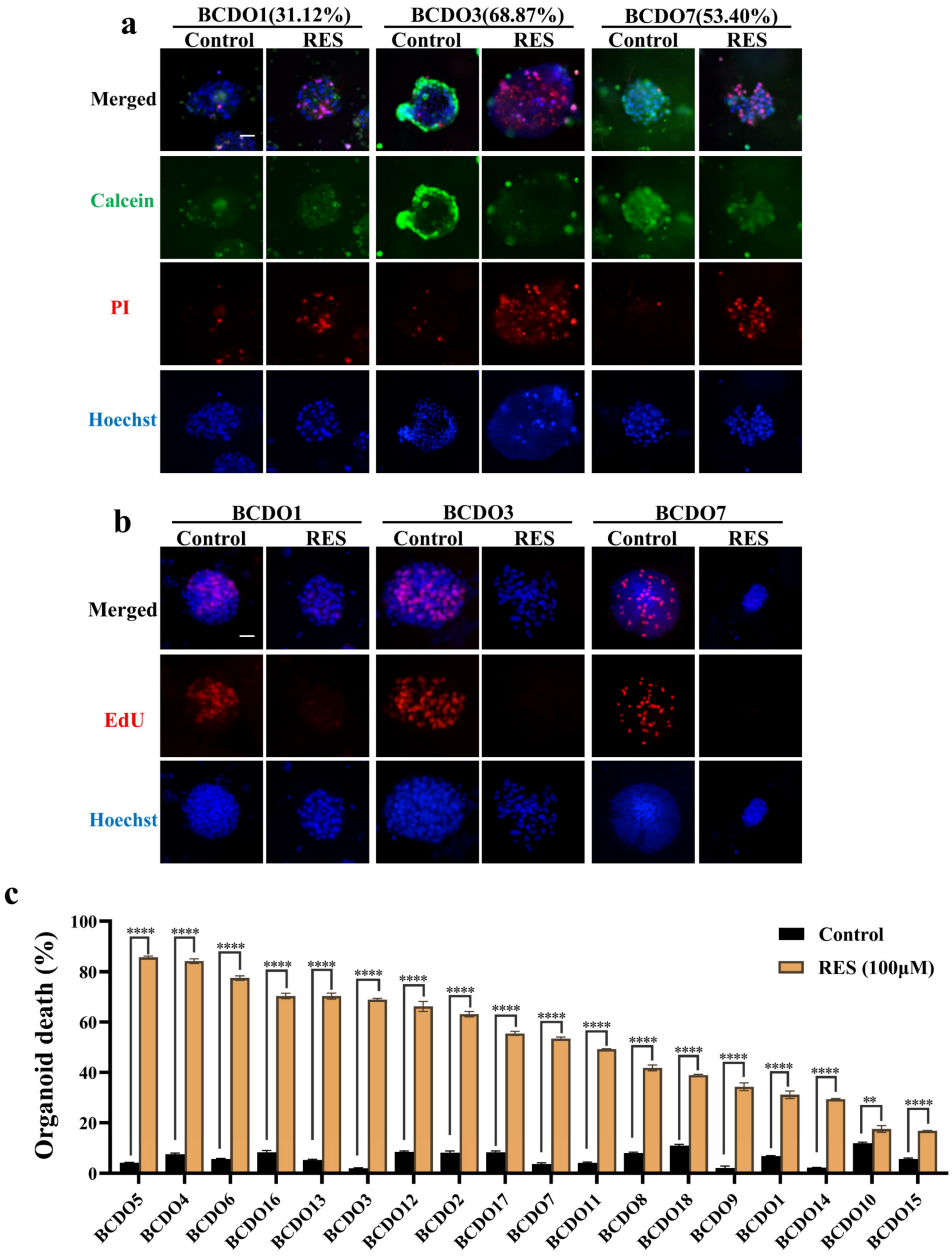
** Evaluation of RES sensitivity of 18 BCDO cases.** (a) The images of viable cells (green) and unviable cells (red) by Calcein/PI staining in BCDOs with 0/100 μM RES treatment for 96h. The values in parentheses after BCDO represent the percentage of dead cells relative to the total number of cells (dead cells /total cells *100%). The scale bar: 50 μm. (b) The natural mortality and RES-caused mortality of 18 cases of BCDOs were shown in the histogram. **, P<0.01; ****<0.0001. Each data point represents the means ±SD (n=3) of independent experiments. (c) The images of proliferative cells by EdU staining in BCDOs with 0/100 μM RES treatment for 96h.

The 18 cases of BCDOs were treated with 100 μM RES for 96h. RES treatment caused extensive intra-organoid cell death and structural disintegration of sensitive BCDOs, while the insensitive ones remained intact and stable (Figure 3a). In accordance, growth suppression was evidenced in RES-sensitive cases by EdU cell proliferation assay (Figure 3b). The results of the cell viability assay revealed that of the 18 cases checked, 10 cases showed over 50% intra-BCDO cell death rates, while the remaining 8 cases showed less than 50% (Table S3 and Figure 3c).

### RES effects on mitochondria fluorescence and ROS levels

**Figure 4:**
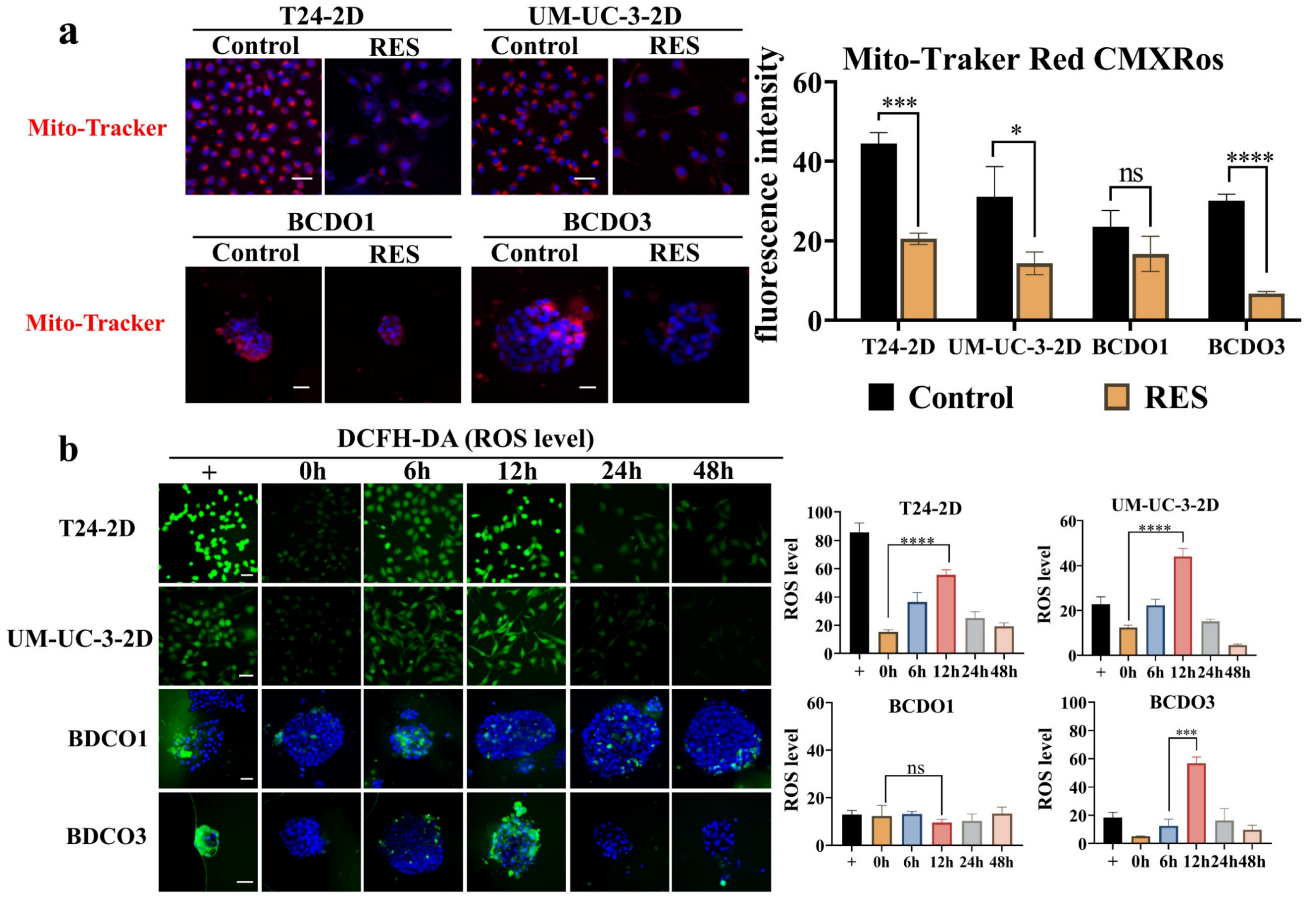
** RES induced mitochondrial dysfunction and oxidative stress**. (a) the images of Mito-Tracker Red CMXRos staining (Left) and the red fluorescence intensity analysis (Right) of the 2D cultured T24 and UM-UC-3 cells and BCDOs (BCDO1: RES-insensitive; BCDO3: RES-sensitive) with or without 100 μM RES treatment for 96h. *, P <0.05; ns, P >0.05. (b) the graphic of the cells and BCDOs treated with 100 μM RES for 0h, 6h, 12h, 24h, 48h by DCFH-DA staining and the ROS levels analysis. ***, P <0.001. The scale bar: 50 μm.

The intensity of the red fluorescent signal reflects the bioactivity and membrane potential of the mitochondria.RES treatment (100 μM) significantly reduced fluorescence intensity in T24 and UM-UC-3 cells, as well as in RES-sensitive BCDOs (P < 0.0001), but not in RES-insensitive BCDOs (P > 0.05) (Figure 4a and Figure S2). ROS levels in RES-treated T24, UM-UC-3 cells, and RES-sensitive BCDOs increased significantly at 6h, peaked at 12h (P < 0.0001; P<0.001; P<0.001), and then declined over time. No significant change in Ros levels was observed in RES-insensitive BCDOs (Figure 4b and Figure S3). These results suggest that RES treatment may affect mitochondrial membrane potential and induce oxidative stress in sensitive cell lines.

### RES induced peroxidation and ferroptosis of BC cells

**Figure 5:**
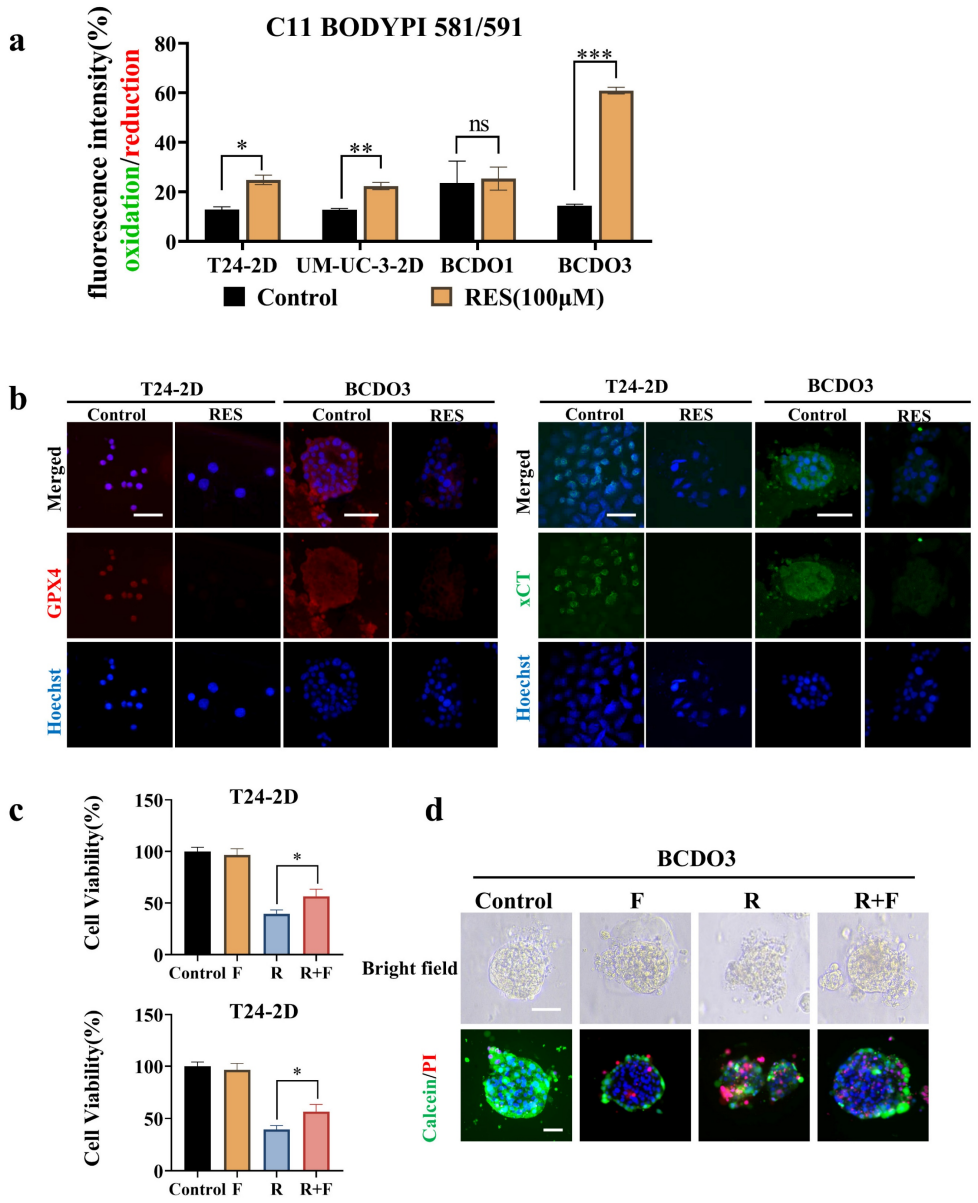
** RES induced ferroptosis and autophagy in BC cells.** (a) RES promotes lipid peroxidation in BC cells, but not in RES-insensitive BCDOs, and the histogram shows the ratio of Green (Oxidation)/Red (Reduction) after semi-quantitative analysis of the fluorescence intensity. *, P <0.05. (b) Immunofluorescent labeling of GPX4 and xCT in T24-2D and BCDOs treated with 0/100 μM RES. (c) The cell viability of T24 by using 0, F (0.5 μM), R (100 μM), R+F for 48h and the and organoid death (%) by using 0, F (0.5 μM), R (100 μM), R+F for 96h. F: ferrostatin-1; R: resveratrol. (d) The images of morphological and live/dead fluorescence of BCDOs after treating with 0, F, R, R+F. The scale bar: 50 μm.

Lipid peroxidation is the key link and characteristic phenomenon leading to ferroptosis 32. The impact of RES on the iron-driven cell death pathway was evaluated by using a C11BODIPY 589/591 lipid peroxidation probe. Fluorescent images showed that red fluorescent intensity (reduction state) was decreased and green fluorescent intensity (oxidation state) increased in 2D cultured T24 and UM-UC-3 cells after 100 μM RES treatment for 24h. Significant lipid peroxidation was observed in RES-sensitive BCDOs rather than RES-insensitive at 48h time point of 100 μM RES treatment. A detailed description of lipid peroxidation statuses of all experimental groups was summarized in the form of fluorescent intensity ratio (Green/Red; Oxidation/Reduction) in Figure 5a and Figure S4. Meanwhile, the expression of GPX4 (resistance to lipid oxidation) and xCT (an important transporter for synthesis of GSH) was down-regulated in T24 and RES-sensitive BCDOs treated with 100 μM RES (Figure 5b). Ferroptosis inhibitor ferrostain-1 inhibited 16.95% of T24 cell death (P < 0.05) and 22.57% of BCDOs death (P< 0.0001) caused by RES (Figure 5c). The structure of the R+F group was clearer than that of the R group, and there were fewer dead cells (Figure 5d). Combined with the elevated ROS levels and mitochondrial dysfunction observed in result 4, it could be suggested that RES induced ferroptosis in BC cells.

## Discussion

The anti-BC effects of RES have been documented 8, 20 and shown again by our findings from 2D cultured T24 and UM-UC-3 cells. However, the majority of the data so far available are obtained from the established human BC cell lines and/or their animal xenograft models. Because the cancer cell lines currently in use have undergone many rounds of passages, they eventually fail to accurately capture the actual biological properties of their original cancer tissues. Therefore, potential bias exists when using the results collected from those cell lines to interpret clinical issues such as drug sensitivity and therapeutic outcomes of individual cases 33. These problems can be largely avoided using patient-derived organoids (PDOs) because PDOs maintain the basic pathological and molecular features of their parental tumors. For this reason, PDOs have been increasingly used as an ex vivo therapeutic platform and therefore employed in the current study.

Our drug sensitivity assay revealed that the two BC cell lines (T24 and UM-UC-3) were sensitive to RES, and the IC_50_ of the drug was around 100 μM at a 48-hour time point; the cell spheroids derived from the two cell lines showed similar sensitivity trend as their 2D cultured counterparts but required longer time (96 hours). This difference may be due to the sophisticated 3D structure of the spheroids and the presence of Matrigel around them. We therefore used 100 μM RES as working concentration to treat the BCDOs for 96 hours. It was found that 10 out of 18 BCDOs (55.56%) were sensitive to RES in the intra-organoid cell death rates from 53.4% to 85.64%. The remaining 8 BCDO cases showed limited responsiveness to RES. These results suggest the potential therapeutic values of RES in BC treatment and, meanwhile, the presence of inter-BC difference in RES sensitivity. Although our preliminary analyses did not reveal significant associations between RES response and clinical characteristics such as muscular invasion or TNM staging, this may be due to the limited sample size in the current study. Currently, we are exploring the underlying reason(s) of the different response of BCs to RES, which may provide deeper insights into the mechanisms driving RES sensitivity.

It has been recognized that RES can cause cancer cell death in different modes 13. For instance, it induces apoptosis of breast cancer cells 34, commits non-small cell lung cancer cells to autophagic death 35, and brings colorectal cancer cells to ferroptosis 36. RES is also able to induce apoptosis of BC cells and their transplanted animal models 10. The above findings implicate that RES via its multifaceted biological activities exerts anti-cancer effects by opening more than one cell death routes. However, no datum has been available to show the bladder cancer patterns of cell death in an RES-treated experimental system. Addressing this issue would be helpful to explain the reason for the better anticancer efficacy of this polyphenol compound than that of conventional chemotherapeutic drugs 37. Excessive oxidative stress can lead to the damage of biomolecules (proteins, lipids, RNA, and DNA) inside the cells, resulting in cell senescence and death 18, 38.

Because the anti-cancer dose (100 μM) of RES caused intracellular ROS accumulation in several kinds of cancers including BC cells 39 and oxidative stress can lead the damaged cells to die in different manners, ROS levels in RES-treated BC cells and BCDOs were assessed. The results revealed that ROS levels were elevated in RES-sensitive T24 and UM-UC-3 cells as well as BCDOs, accompanied by extensive cell death upon drug treatment. In contrast, the oxidative status in RES-treated insensitive BCDOs remained stable. These results indicate a close correlation of ROS levels with RES sensitivity of BC cells *in vitro* and *ex vivo*. Because oxidative damage may result in diverse mechanisms leading to irreversible cellular demise, it would be possible that the oxidative stress may trigger different death pathways in RES-treated BC cells.

Mitochondrial dysfunction is a direct result of excessive oxidative stress 40, and mitochondrial dysfunction further leads to the accumulation of ROS inside the cell. Therefore, the statuses of ROS levels and mitochondrial function are closely related to cell survival and drug efficacy. ROS-mediated endoplasmic reticulum stress and mitochondrial damage were involved in RES-induced apoptosis of A549 lung cancer cells 35. ROS accumulation causes intense mitochondrial autophagy of metastatic Hela cancer cells 41. Increased oxidative stress in colon cancer cells can trigger mitochondrial dysfunction and ferroptosis 36. In our experiments, the integrity and biological activity of mitochondria were evaluated using the Mito-Tracker Red CMXRos probe. The mitochondrial activity in RES-treated BCDOs decreased in terms of the decreased red fluorescent intensity accompanied by increased fraction of apoptotic cells, the ferroptosis inhibitor Ferrostatin-1 rescued 16.95% of T24 and 22.57% of intra-BCDO cells from RES-caused death. Meanwhile, lipid peroxidation was detected with the lipid peroxide probe C11, accompanied with GPX4 and xCT downregulation. These evidences suggest that RES is able to induce ferroptosis of BC cells. On the contrary, the above phenomena were not detected in RES-treated but insensitive BCDOs. In this experiment, while our results suggest that RES treatment may affect mitochondrial membrane potential and induce oxidative stress in sensitive cell lines, a conclusion that is also based on previous studies 42, 43, further studies using additional assays, such as JC-1 staining or ATP production measurements, would provide valuable insights to confirm these findings and further elucidate the underlying mechanism. In addition, the rescue rate of the ferroptosis inhibitor was not high, therefore, additional experiments were conducted, including those involving apoptosis inhibitors and autophagy inhibitors. As shown in Figure S5, strong TUNEL green signals were observed in a significant fraction of cells in RES-sensitive BCDOs. The apoptosis inhibitor Z-VAD-FMK only inhibits T24 and intra-BCDO cell death in rates of 10.19% and 11.51%, respectively. Moreover, the upregulation of autophagy-related proteins, LC3α/β and Becin1, was observed in RES-sensitive BCDOs, and autophagy inhibitor Chloroquine reduced the death rates of RES-treated T24 (16.13%) and BCDOs (20.09%), reflecting the presence of RES-enhanced autophagic activity. These supplementary experiments indicate that in the system treated with RES, ferroptosis is present alongside other modes of cell death, which warrants further investigation. These findings suggest that by modulating the interactions among these cell death pathways, RES can achieve a comprehensive cytotoxic effect on BC cells, offering better prospects and specificity compared to single-target chemotherapy drugs. To further enhance the clinical relevance of these findings, future studies should consider incorporating detailed patient medication histories and comorbidities (metabolic or inflammatory conditions) that may influence the activity of ferroptosis or other cell death pathways. Additionally, expanding the sample size will be crucial to uncover potential clinical patterns an validate the observed trends. This approach will help refine the therapeutic potential of RES and ensure its applicability across diverse patient populations.

For BCs, intravesical drug instillation after transurethral resection [44]is a common method to prevent recurrence. This approach of targeted drug delivery to the organ overcomes the low bioavailability 45 issue associated with the clinical use of RES. We previously demonstrated the efficacy and safety of RES intravesical instillation in a mouse model 10 and confirmed this time the favorable therapeutic effect of RES on BCDOS by targeting more than one death-related signaling pathways. The above evidence suggests the potential application of RES in intravesical treatment of BCs. It should be noted that in difference with the findings from the BC cell lines (such as T24 and UM-UC-3), there are still some RES-insensitive BCDO cases. In this context, RES should be used in a personalized manner according to BCDO-based RES sensitivity assays.

## Conclusion

Our current study demonstrates the efficacy of RES against bladder cancer organoids derived from different patients. We found that RES exerts its anti-bladder cancer effect by activating the ferroptosis pathway linked with oxidation-caused damages such as mitochondrial dysfunction, lipid peroxidation, and downregulation of GPX4 and xCT proteins. The supplementary experiments indicated that in bladder cancer cells affected by RES, not only is the ferroptosis pathway activated, but apoptosis and autophagy are also involved. Therefore, RES may activate multiple cell death pathways to induce bladder cancer cell death, further confirming its muti-targeting nature and therapeutic advantages for treating bladder cancer cells. RES would be an adjuvant option in personalized bladder cancer management.

## Supplementary Material

Supplementary figures and tables.
