# Supplementary material for: Resveratrol Induces Oxidative Stress and Downregulates GPX4 and xCT to Activate the Ferroptosis Pathway for Anti-Bladder Cancer Organoids
Source: J Cancer. 2025 Jun 9;16(8):2613–25. doi: 10.7150/jca.109350 (PMC12170997; doi:10.7150/jca.109350)
Supplement: Supplementary file 1 — Supplementary figures and tables. [file jcav16p2613s1.zip › Table S3.docx]

**Table S3. Different effects of RES on bladder cancer organoid for 96h.**

| CODE | Bladder Cancer Organoid Death (%) | | | |
| --- | --- | --- | --- | --- |
|  | Natural mortality | RES(100μM) | Difference Mean ± SEM | Death (%）＞50% |
| BCDO1 | 6.81% | 31.12% | 24.32% ± 0.88% | - |
| BCDO2 | 8.11% | 63.11% | 55.00% ± 0.75% | √ |
| BCDO3 | 2.00% | 68.87% | 66.87% ± 0.28% | √ |
| BCDO4 | 7.55% | 84.13% | 76.58% ± 0.60% | √ |
| BCDO5 | 4.10% | 85.64% | 81.54% ± 0.32% | √ |
| BCDO6 | 5.63% | 77.44% | 71.82%±0. 54% | √ |
| BCDO7 | 3.70% | 53.40% | 49.70%± 0.44% | √ |
| BCDO8 | 8.02% | 41.79% | 33.77% ±0.70% | - |
| BCDO9 | 2.05% | 34.29% | 32.24% ± 1.00% | - |
| BCDO10 | 11.95% | 17.49% | 5.54% ± 0.79% | - |
| BCDO11 | 4.05% | 49.19% | 45.14% ± 0.26% | - |
| BCDO12 | 8.43% | 66.17% | 57.75% ± 1.20% | √ |
| BCDO13 | 5.21% | 70.27% | 65.06% ± 0.75% | √ |
| BCDO14 | 2.24% | 29.39% | 27.15% ± 0.15% | - |
| BCDO15 | 5.62% | 16.83% | 11.22%± 0.27% | - |
| BCDO16 | 8.31% | 70.33% | 62.02% ± 0.76% | √ |
| BCDO17 | 8.31% | 55.45% | 47.14% ± 0.58% | √ |
| BCDO18 | 10.93% | 38.90% | 27.98% ± 0.36% | - |

RES: Resveratrol.
