# Supplementary material for: Resveratrol Induces Oxidative Stress and Downregulates GPX4 and xCT to Activate the Ferroptosis Pathway for Anti-Bladder Cancer Organoids
Source: J Cancer. 2025 Jun 9;16(8):2613–25. doi: 10.7150/jca.109350 (PMC12170997; doi:10.7150/jca.109350)
Supplement: Supplementary file 1 — Supplementary figures and tables. [file jcav16p2613s1.zip › FigureS1-S6.pdf]

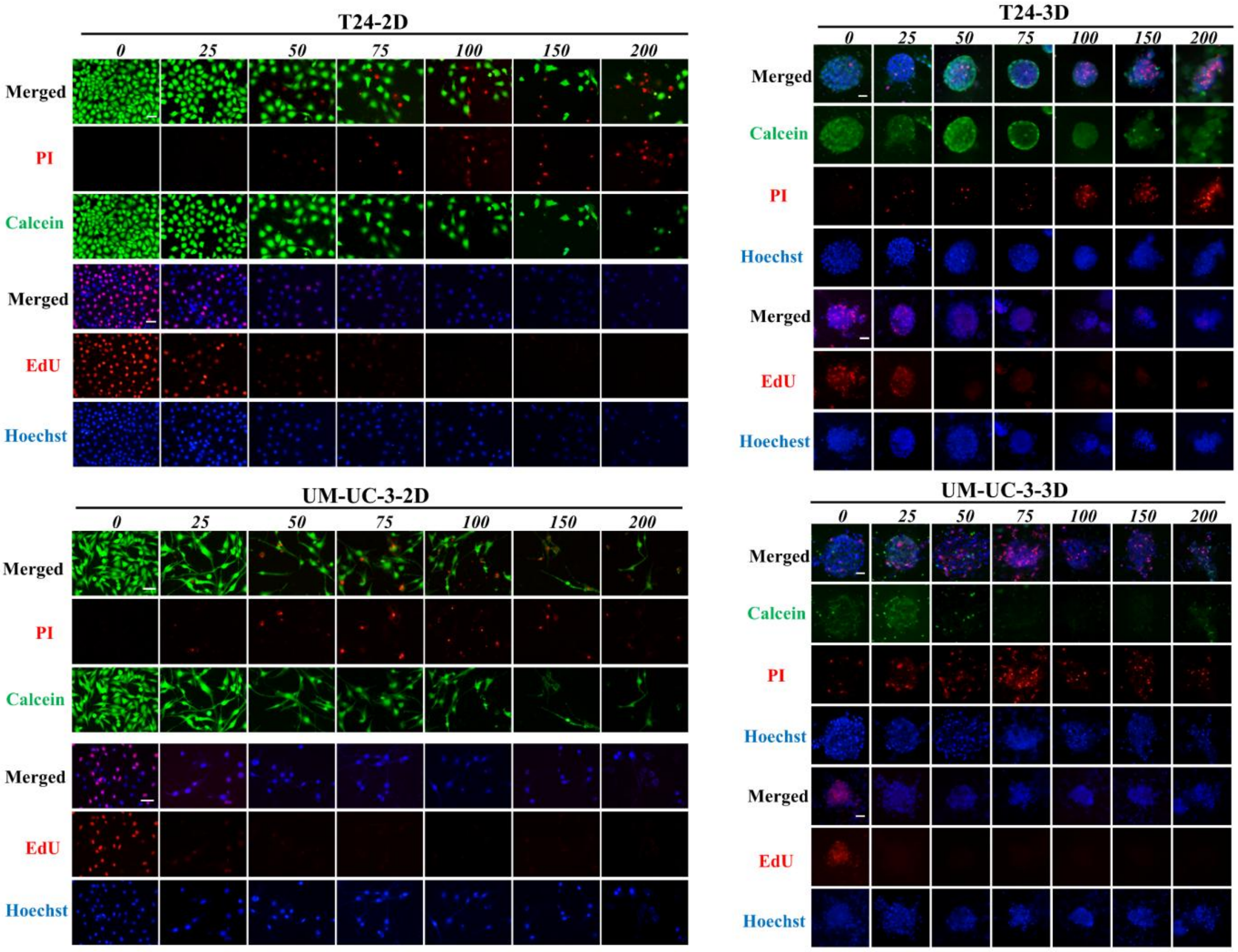

Figure S1. The images of Calcein/Pl, EdU, and Hoechst staining of the T24 and UM-UC-3 cells and spheroids with different concentrations (0,25,50, 100, 150, 200 μM) RES treatment for 48h or 96h. The scale bar: 50 μm.

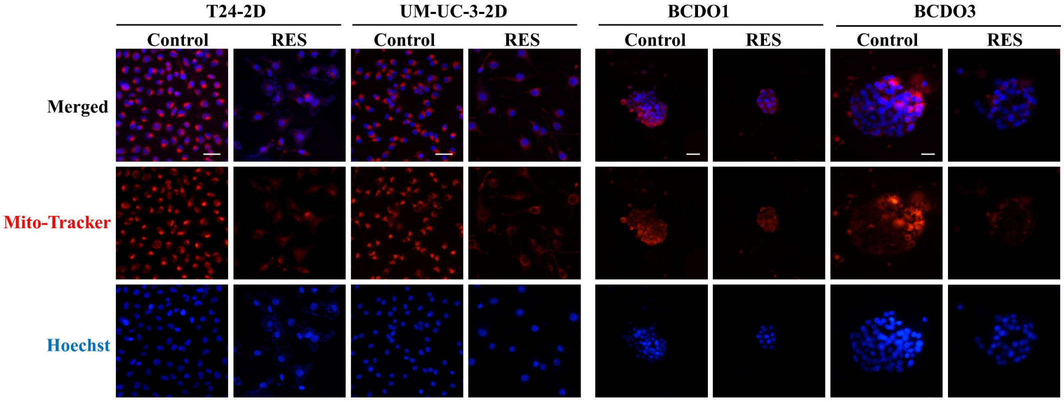

Figure S2. The images of Mito-Tracker Red CMRos and Hoechst staining of the 2D cultured T24 and UM-UC-3 cells for 48h as well as BCDO1 (RES-insensitive) and BCDO3 (RES-sensitive) with or without 100 μM RES treatment for 96h. The scale bar: 50 μm.

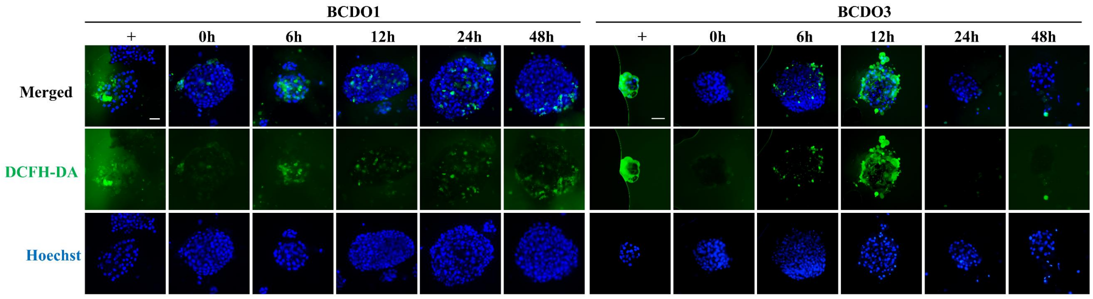

Figure S3. The graphic of T24 and UM-UC-3 cells and BCDOs treated with 100 μM RES for 0h, 6h, 12h, 24h, and 48h by DCFH-DA and Hoechst staining.

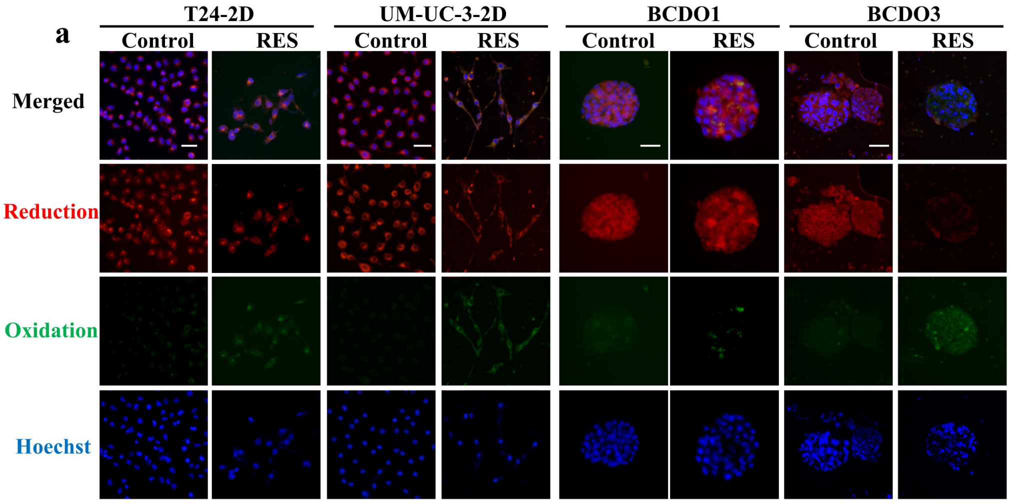

Figure S4. The images of C11 BODIPY 589/591 staining of T24 and UM-UC-3 cells with 0/100 μM RES for 24h and BCDOs for 96h. Here, RES promotes lipid peroxidation in BC cells, but not in RES-insensitive BCDOs. The scale bar: 50 μm.

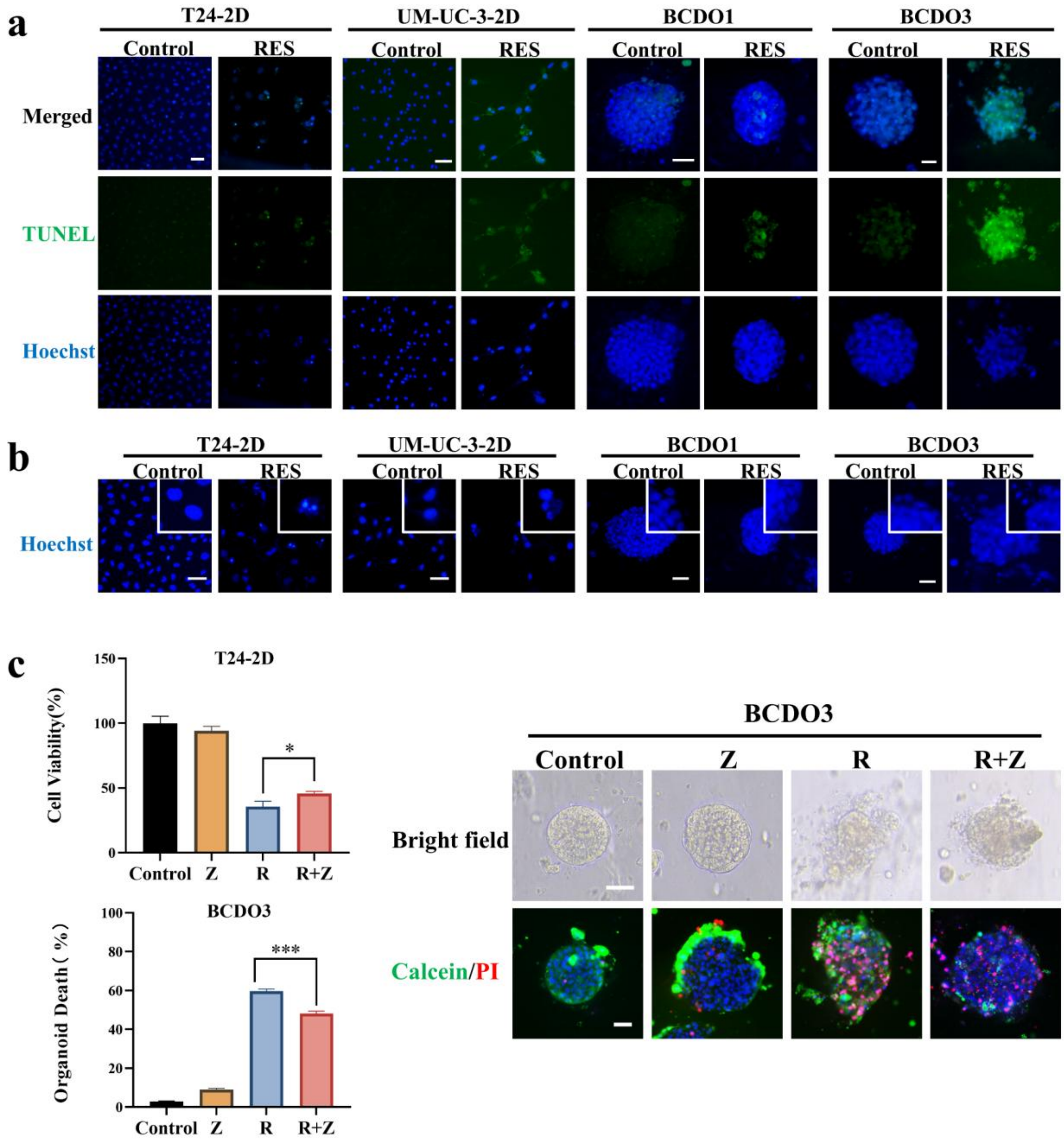

Figure S5. Resveratrol induced apoptosis in BC cells. (a) TUNEL illustration of RES induced apoptosis in 2D cultured of T24 and UM-UC-3 cells, and RES induced apoptosis in RES-sensitive BCDO3 but not insensitive BCDO1. (b) images of Hoechst staining in 2D cultured T24 and UM-UC-3 cells treated by 100 μM for 48h and BCDOs for 96h. (c) The cell viability of T24 by using 0、Z (0.5 μM), R (100 μM), R+Z for 48h and the organoid death (%) by using 0, Z (0.5 μM) for 96h, R (100 μM), R+Z for 96h. Z: Z-VAD-FMK; R: resveratrol. (d) The images of bright field morphological and live/dead fluorescence of BCDOs (Control、Z、R、R+Z). The scale bar: 50 μm.

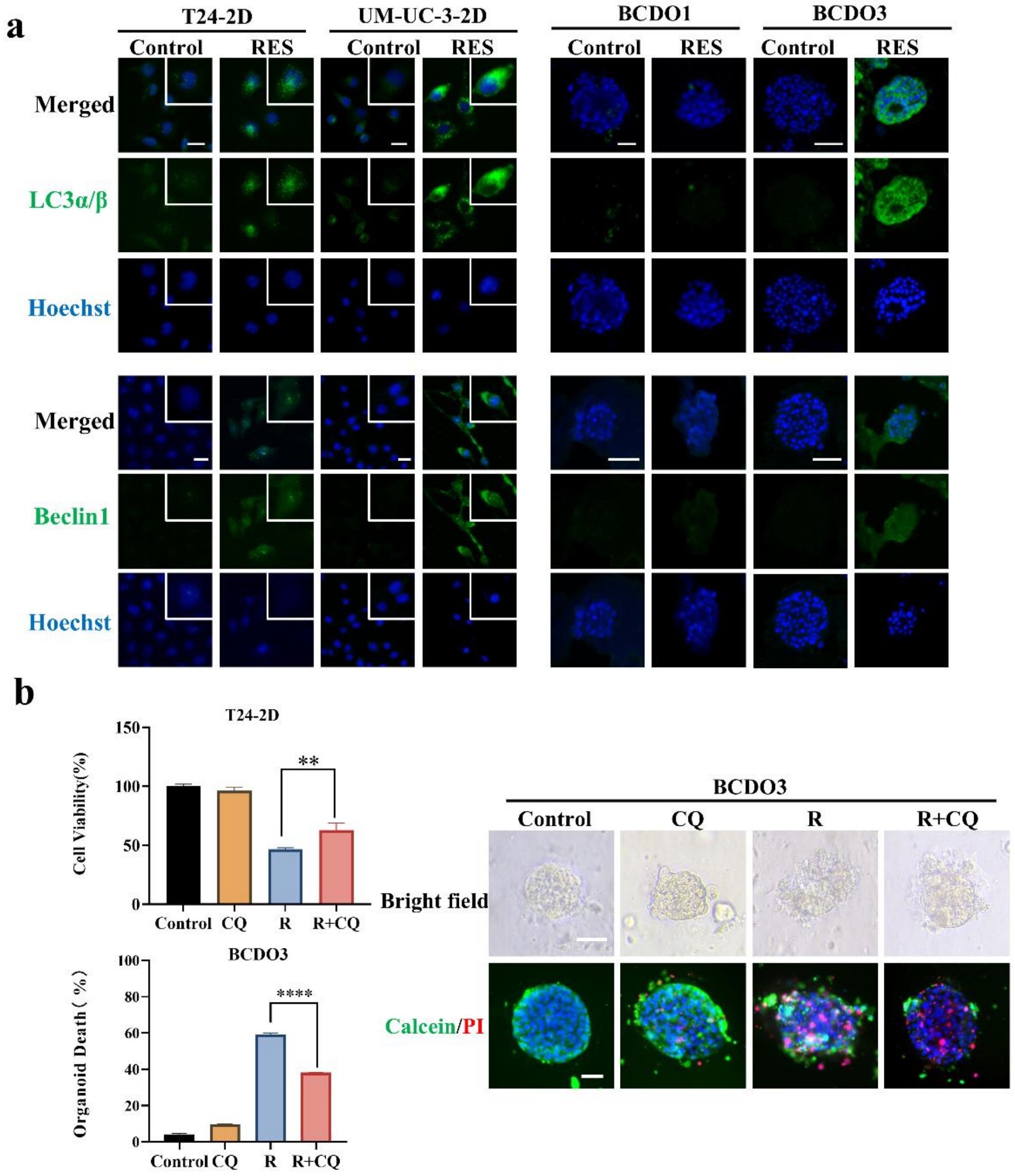

Figure S6. Resveratrol induced autophagy in BC cells. (a) Altered expression of LC3α/β, and Beclin1 in T24 and UM-UC-3 cells with 0/100 μM RES treatment for 48h. LC3α/β, and Beclin1 expression was altered in RES-sensitive BCDO3 but not in RES-insensitive BCDO1. (b) The cell viability of T24 by using 0, CQ (0.5 μM), R (100 μM), R+CQ for 48h and the and organoid death (%) by using 0, F (0.5 μM), R (100 μM), R+CQ for 96h. CQ: Chloroquine; R: resveratrol. The images of morphological changes and cell activity of BCDOs after 0, CQ, R, R+CQ. The scale bar: 50 μm.
