# Supplementary material for: Resveratrol Induces Oxidative Stress and Downregulates GPX4 and xCT to Activate the Ferroptosis Pathway for Anti-Bladder Cancer Organoids
Source: J Cancer. 2025 Jun 9;16(8):2613–25. doi: 10.7150/jca.109350 (PMC12170997; doi:10.7150/jca.109350)
Supplement: Supplementary file 1 — Supplementary figures and tables. [file jcav16p2613s1.zip › Table S1.docx]

**Table S1. Clinical information of 18 patients corresponding to BCDOs.**

| **Patient code** | **Gender** | **Age** | **TNM** | **Histology type** | **Grade** | **Muscular invasion** | **Surgical method** | **IHC** | | | | |
| --- | --- | --- | --- | --- | --- | --- | --- | --- | --- | --- | --- | --- |
|  |  |  |  |  |  |  |  | **GATA-3** | **CK7** | **P63** | **KI67** | **P53** |
| BCDO1 | male | 64 | T2N0M0 | Urothelial carcinoma | high grade | √ | RC | + | + | + | / | 20% |
| BCDO2 | male | 69 | T3aN0M0 | Urothelial carcinoma | high grade | NS | TURBT | + | + | / | 80% | / |
| BCDO3 | male | 45 | TaN0M0 | Papillary urothelial carcinoma | high grade | / | TURBT | + | / | / | 50% | √, wide type |
| BCDO4 | male | 38 | TaN0M0 | Papillary urothelial carcinoma | high grade | NS | TURBT | / | / | / | 25% | 20% |
| BCDO5 | male | 62 | TaN0M0 | Papillary urothelial carcinoma | high grade | / | TURBT | + | / | / | 90% | √ |
| BCDO6 | male | 57 | TaN0M0 | Papillary urothelial carcinoma | high grade | / | TURBT | / | / | / | 15% | √ |
| BCDO7 | male | 63 | TaN0M0 | Papillary urothelial carcinoma | high grade | √ | RC | / | + | + | 10% | 10% |
| BCDO8 | male | 59 | TaN0M0 | Papillary urothelial carcinoma | high grade | √ | RC | / | + | + | 20% | 55% |
| BCDO9 | male | 56 | TaN0M0 | Papillary urothelial carcinoma | high grade | / | TURBT | / | / | / | 30% | 80% |
| BCDO10 | male | 71 | TaN0M0 | Papillary urothelial carcinoma | high grade | / | TURBT | / | + | / | 35% | 20% |
| BCDO11 | male | 65 | T2N1M1 | Urothelial carcinoma | high grade | √ | TURBT | + | / | / | 30% | / |
| BCDO12 | female | 73 | T1N0M0 | Urothelial carcinoma | high grade | NS | TURBT | + | / | + | 50% | √, mutant type |
| BCDO13 | male | 63 | T3bN0M0 | Urothelial carcinoma | high grade | √ | RC | + | + | + | 30% | 60% |
| BCDO14 | male | 80 | TaN0M0 | Papillary urothelial carcinoma | high grade | √ | TURBT | / | + | / | 15% | 60% |
| BCDO15 | male | 71 | T2N0M0 | Urothelial carcinoma | high grade | √ | TURBT | + | + | / | 90% | √ |
| BCDO16 | male | 72 | T3bNxM1 | Urothelial carcinoma | high grade | / | TURBT | + | / | / | 80% | 70% |
| BCDO17 | male | 71 | T1N0M0 | Papillary urothelial carcinoma | high grade | / | TURBT | + | / | / | 60% | 40% |
| BCDO18 | male | 74 | TaN0M0 | Papillary urothelial carcinoma | high grade | / | TURBT | + | / | / | 8% | 35% |

**BCDO:** bladder cancer derived organoid; **NS:** not seen muscular layer; **RC:** Radical Cystectomy; **TURBT:** Transurethral Resection of Bladder Tumor; **IHC:** Immunohistochemistry;**√:**have muscular invasion; **/:** non-muscular invasion or not expression; **+：**there is a positive expression; **%:** How much specific expression
