# Supplementary material for: Resveratrol Induces Oxidative Stress and Downregulates GPX4 and xCT to Activate the Ferroptosis Pathway for Anti-Bladder Cancer Organoids
Source: J Cancer. 2025 Jun 9;16(8):2613–25. doi: 10.7150/jca.109350 (PMC12170997; doi:10.7150/jca.109350)
Supplement: Supplementary file 1 — Supplementary figures and tables. [file jcav16p2613s1.zip › Table S2.docx]

**Table S2. RES IC_50_ of 2D-cultured T24 and UM-UC-3 cells and their spheroids.**

| IC_50_（RES） | T24 | UM-UC-3 |
| --- | --- | --- |
| 2D (48h） | 106.77±3.59 (μM） | 94.17±4.56 (μM） |
| 3D (48h） | 141.3±4.13 (μM） | 150.6±4.85 (μM） |
| 3D (96h） | 103.54±2.07 (μM） | 89.47±5.73 (μM） |

RES: Resveratrol, each point presented as mean ± SD (n=3).
